# A Generalized Information-Theoretic Approach for Bounding the Number of Independent Sets in Bipartite Graphs

**DOI:** 10.3390/e23030270

**Published:** 2021-02-25

**Authors:** Igal Sason

**Affiliations:** Department of Electrical Engineering, Technion—Israel Institute of Technology, Haifa 3200003, Israel; sason@ee.technion.ac.il; Tel.: +972-4-8294699

**Keywords:** Shannon entropy, Shearer’s lemma, counting, independent sets, graphs

## Abstract

This paper studies the problem of upper bounding the number of independent sets in a graph, expressed in terms of its degree distribution. For bipartite regular graphs, Kahn (2001) established a tight upper bound using an information-theoretic approach, and he also conjectured an upper bound for general graphs. His conjectured bound was recently proved by Sah et al. (2019), using different techniques not involving information theory. The main contribution of this work is the extension of Kahn’s information-theoretic proof technique to handle irregular bipartite graphs. In particular, when the bipartite graph is regular on one side, but may be irregular on the other, the extended entropy-based proof technique yields the same bound as was conjectured by Kahn (2001) and proved by Sah et al. (2019).

## 1. Introduction

The Shannon entropy and other classical information measures serve as a powerful tool in various combinatorial and graph-theoretic applications (see, e.g., [1,2,3,4,5,6,7,8,9,10,11,12,13,14,15,16,17,18,19,20]), such as the method of types, applications of Shearer’s lemma, sub- and supermodularity properties of information measures and their applications, entropy-based proofs of Moore bound for irregular graphs, Bregman’s theorem on the permanent of square matrices with binary entries, and a discrepancy theorem by Spencer.

The enumeration of discrete structures that satisfy certain local constraints, and particularly the enumeration of independent sets in graphs, is of interest in discrete mathematics. Many important structures can be modeled by independent sets in a graph, i.e., subsets of vertices in a graph where none of them are connected by an edge. For example, if a graph models some kind of incompatibility, then an independent set in this graph represents a mutually compatible collection. Upper bounding the number of independent sets in a regular graph was motivated in [21] by a conjecture which has several applications in combinatorial group theory. A survey paper on upper bounding the number of independent sets in graphs, along with some of their applications, is provided in [22]. The problem of counting independent sets in graphs received, in general, significant attention in the literature on discrete mathematics over the last three decades, and also in the information theory literature ([13,14]).

A tight upper bound on the number of independent sets in finite and undirected general graphs was proved in the special setting of bipartite regular graphs in [11], and it was conjectured to hold for general (irregular) graphs (2001, see Conjecture 4.2 in [11]). A decade later (2010), it was extended in [23] to regular graphs (that are not necessarily bipartite); a year later (2011), it was proved in [24] for graphs with a small maximal degree (up to 5). Finally, this conjecture was recently (2019) proved in general [25], by utilizing a new approach. The reader is referred to [26] for an announcement on the solution of this conjecture as a frustrating combinatorial problem for two decades, along with the history and ramifications of this problem, and some reflections of the authors on their work in [25].

The recently introduced proof of the conjecture for *general* undirected graphs [25] uses an induction on the number of vertices in a graph, and it obtains a recurrence inequality whose derivation involves some judicious applications of Hölder’s inequality (see Sections 2 and 4 in [25]). The work in [25] proved, for the first time, Conjecture 4.2 in [11] by using an interesting approach which is unrelated to information theory. The possibility of generalizing the information-theoretic proof in [11] to irregular bipartite graphs was left in [25] as an open issue. It should be noted that by proving Kahn’s conjecture for irregular bipartite graphs, this readily enables the extension of the proof to general undirected graphs (by invoking Zhao’s inequality, see Lemma 3 in [24]).

The main contribution of this work is the extension of Kahn’s information-theoretic proof technique for bipartite regular graphs [11] to handle irregular bipartite graphs. In particular, when the bipartite graph is regular on one side, but may be irregular on the other, the extended entropy-based proof technique yields the same bound that was conjectured by Kahn [11] and proved by Sah et al. [23].

The structure of the paper is as follows: Section 2 provides preliminaries and notations that are essential for the analysis in this paper. Section 3 explains (in more detail) the scientific merit and contributions of the present work; for the sake of causal presentation, we provide these explanations after Section 2. Section 4 and Section 5 are the core of this work.

## 2. Preliminaries and Notation

In this section, we provide the notation and preliminary material which are essential for the presentation in this paper.

### 2.1. Notation and Basic Properties of the Entropy

The following notations are used in the present paper:

- N≜{1,2,…} denotes the set of natural numbers;
- $X^{n}≜\left(X_{1},\ldots X_{n}\right)$ denotes an *n*-dimensional random vector of discrete random variables, having a joint probability mass function (PMF) that is denoted by PXnXYZ;
- For every n∈N, [1:n]≜{1,…,n};
- $X_{S}≜{\left(X_{i}\right)}_{i\in S}$ is a random vector for an arbitrary nonempty subset S⊆[1:n]; if S=∅, then conditioning on $X_{S}$ is void. Note that $X^{n}=X_{\left[1:n\right]}$, though $X^{n}$ is a commonly used notation;
- 𝟙{E} denotes the indicator of an event *E*; i.e., it is equal to 1 if this event is satisfied, and it is zero otherwise;
- Let *X* be a discrete random variable that takes its values on a set X, and let PXXYZ be the PMF of *X*. The *Shannon entropy* of *X* is given by
  (1) H(X)≜−∑x∈XPXXYZ(x)logPXXYZ(x),
  where throughout this paper, we take all logarithms to base 2;
- For p∈[0,1],
  (2) $$\begin{array}{c} H_{b}\left(p\right)≜-p\log p-\left(1-p\right)\log\left(1-p\right), \end{array}$$
  where $H_{b}\left(\cdot \right)$ is the *binary entropy function*. By continuous extension, the convention 0log0=0 is used;
- Let *X* and *Y* be discrete random variables with a joint PMF PXYXYZ, and a conditional PMF of *X* given *Y* that is denoted by PXXYZ|YXYZ. Let *X* and *Y* take their values in the sets X and Y, respectively. The *conditional entropy* of *X* given *Y* is defined as
  (3)H(X|Y)≜−∑(x,y)∈X×YPXYXYZ(x,y)logPXXYZ|YXYZ(x|y)(4)=∑y∈YPYXYZ(y)H(X|Y=y),
  and
  (5) $$\begin{array}{cc} H(X|\;Y) & =H(X,Y)-H(Y). \end{array}$$

This paper relies on the following basic properties of the Shannon entropy:

- Entropies and conditional entropies of discrete random variables (or vectors) are nonnegative;
- If X is a finite set, then
  (6) $$\begin{array}{c} H(X)\leq \log|X|, \end{array}$$
  with equality in (6) if and only if *X* is equiprobable over the set X;
- Conditioning cannot increase the entropy, i.e.,
  (7) $$\begin{array}{c} H(X|\;Y)\leq H(X), \end{array}$$
  with equality in (7) if and only if *X* and *Y* are independent;
- Generalizing (5) to *n*-dimensional random vectors gives the chain rule for the Shannon entropy:
  (8)H(Xn)=H(X1)+H(X2|X1)+…+H(Xn|X1,…,Xn−1)(9)=∑i=1nH(Xi|Xi−1).
- The following *subadditivity property of the entropy* is implied by (7) and (9)
  (10) $$\begin{array}{c} H\left(X^{n}\right)\leq \sum_{i=1}^{n}H\left(X_{i}\right), \end{array}$$
  with equality in (10) if and only if $X_{1},\ldots ,X_{n}$ are independent random variables.

### 2.2. Shearer’s Lemma

Shearer’s lemma extends the subadditivity property (10) of the entropy. Due to its simplicity and usefulness in this paper (and elsewhere), we state and prove this lemma here.

**Proposition** **1** (Shearer’s Lemma, *[2]). Let $X_{1},\ldots ,X_{n}$ be discrete random variables, and let the sets $S_{1},\ldots ,S_{m}\subseteq \left[1:n\right]$ include every element i∈[1:n] in at least k≥1 of these subsets. Then,*

(11) $$\begin{array}{c} kH\left(X^{n}\right)\leq \sum_{j=1}^{m}H\left(X_{S_{j}}\right). \end{array}$$

As a special case of (11), setting $S_{i}≜\left\{i\right\}$ as singletons for all i∈[1:n] gives (10) by having k=1 and m=n.

**Proof.**  Let $S=\{i_{1},\ldots ,i_{\ell }\}$ with $1\leq i_{1}<\ldots <i_{\ell }\leq n$. By invoking the chain rule in this order,

H(XS)=H(Xi1)+H(Xi2|Xi1)+…+H(Xiℓ|Xi1,…,Xiℓ−1)≥∑i∈SH(Xi|Xi−1)(12)=∑i=1n𝟙{i∈S}H(Xi|Xi−1),

where the last inequality holds since an additional conditioning cannot increase the entropy (i.e., H(X|Y,Z)≤H(X|Y) for all X,Y and *Z*). By assumption, for i∈[1:n],

(13) $$\begin{array}{c} \sum_{j=1}^{m}𝟙\left\{i\in S_{j}\right\}\geq k, \end{array}$$

since the number of subsets ${\left\{S_{j}\right\}}_{j=1}^{m}$ that include *i* as an element is at least *k*. Consequently, it follows that

(14)∑j=1mH(XSj)≥∑j=1m∑i=1n𝟙{i∈Sj}H(Xi|Xi−1)(15)=∑i=1n∑j=1m𝟙{i∈Sj}H(Xi|Xi−1)(16)≥k∑i=1nH(Xi|Xi−1)(17)=kH(Xn),

where (14) follows from (12); (15) holds by interchanging the order of summation; (16) holds by (13); (17) holds by the chain rule (8). □

**Remark** **1.** *Inequality* (11) *holds even if the sets $S_{1},\ldots ,S_{m}$ are not necessarily included in [1:n]. To verify this, define the subsets $S_{j}^{\prime }≜S_{j}\cap \left[1:n\right]$ for all j∈[1:m]. The subsets $S_{1}^{\prime },\ldots ,S_{m}^{\prime }$ are all included in [1:n], and every element i∈[1:n] continues to be included in at least k≥1 of these subsets. Hence, Proposition 1 can be applied to the subsets $S_{1}^{\prime },\ldots ,S_{m}^{\prime }$. By the monotonicity property of the entropy, the inclusion $S_{j}^{\prime }\subseteq S_{j}$ implies that $H\left(X_{S_{j}^{\prime }}\right)\leq H\left(X_{S_{j}}\right)$ for all j∈[1:m], which then yields the satisfiability of* (11).

**Remark** **2.** *A generalized inequality which extends both Shearer’s lemma and Han’s inequality is provided in Proposition 1 in [14].*

Shearer’s lemma and some of its variants (see [7,9]) have successfully been applied on various occasions (see, e.g., [7,8,9,11,12,19]). Shearer’s lemma is also instrumental in this paper.

### 2.3. Graphs, Independent Sets, and Tensor Products

Let *G* be an undirected graph, and let V(G) and E(G) denote, respectively, the sets of vertices and edges in *G*.

A graph *G* is called *d-regular* if the degree of all the vertices in V(G) is equal to *d*. Otherwise, if the graph *G* is not *d*-regular for some d∈N, then *G* is an *irregular* graph.

A graph *G* is called *bipartite* if it has two types of vertices, and the edges cannot connect vertices of the same type; we refer to the two types of vertices of a bipartite graph *G* as left and right vertices.

A graph *G* is called *complete* if every vertex v∈V(G) is connected to all the other vertices in V(G)\{v} (and not to itself); similarly, a bipartite graph is called complete if every vertex is connected to all the vertices of the other type in the graph. A complete (d−1)-regular graph is denoted by $K_{d}$, having a number of vertices $\left|V(K_{d})\right|=d$, and a number of edges $\left|E(K_{d})\right|=\frac{1}{2}\;d\left(d-1\right)$. Likewise, a complete *d*-regular bipartite graph is denoted by $K_{d,d}$, having a number of vertices $\left|V(K_{d,d})\right|=2d$ (i.e., *d* vertices of each of the two types), and a number of edges $\left|E(K_{d,d})\right|=d^{2}$.

An *independent set* of an undirected graph *G* is a subset of its vertices such that none of the vertices in this subset are adjacent (i.e., none of them are joined by an edge). Let L(G) denote the set of all the independent sets in *G*, and let $\left|L(G)\right|$ denote the number of independent sets in *G*. Similarly to [8,11,13,14,19,21,22,23,24,25] (and references therein), our work considers the question of how many independent sets *G* can have.

The *tensor product* G×H of two undirected graphs *G* and *H* is a graph such that the following holds:

- The vertex set of G×H is the Cartesian product V(G)×V(H);
- Two vertices $\left(g,h\right),\left(g^{\prime },h^{\prime }\right)\in V\left(G\times H\right)$ are adjacent if and only if *g* is adjacent to $g^{\prime }$, and *h* is adjacent to $h^{\prime }$, i.e., $\left(g,g^{\prime })\in E(G\right)$ and $\left(h,h^{\prime }\right)\in E\left(H\right)$. This is denoted by $\left(\left(g,h\right),\left(g^{\prime },h^{\prime }\right)\right)\in E\left(G\times H\right)$.

In general, the following identities hold:

(18) $$\begin{array}{cc} \; & \left|V\left(G\times H\right)\right|=\left|V(G)\right|\;\left|V(H)\right|, \end{array}$$

(19) $$\begin{array}{cc} \; & \left|E\left(G\times H\right)\right|=2\;\left|E(G)\right|\;\left|E(H)\right|. \end{array}$$

By the definition of a complete *d*-regular graph $K_{d}$, the graph $K_{2}$ is specialized to two vertices that are connected by an edge. Let us label the two vertices in $K_{2}$ by 0 and 1. For a graph *G*, the tensor product $G\times K_{2}$ is a bipartite graph, called the *bipartite double cover* of *G*, where the set of vertices in $G\times K_{2}$ is given by

(20) $$\begin{array}{c} V(G\times K_{2})=\left\{(v,i):v\in V(G),\;i\in \{0,1\}\right\}, \end{array}$$

and its set of edges is given by

(21) $$\begin{array}{c} E(G\times K_{2})=\left\{\left((u,0),(v,1)\right):\left(u,v\right)\in E(G)\right\}. \end{array}$$

Every edge e=(u,v)∈E(G) is mapped into the two edges $\left((u,0),(v,1)\right)\in E(G\times K_{2})$ and $\left((v,0),(u,1)\right)\in E(G\times K_{2})$ (since the graph *G* is undirected). This implies that the numbers of vertices and edges in $G\times K_{2}$ are doubled in comparison to their respective numbers in *G*; moreover, every edge in *G*, which connects a pair of vertices of specified degrees, is mapped onto two edges in $G\times K_{2}$, where each of these two edges connects a pair of vertices of the same specified degrees.

### 2.4. Upper Bounds on the Number of Independent Sets

The present subsection introduces the relevant results to this paper. The next theorem provides a tight upper bound on the number of independent sets in bipartite regular graphs, and its derivation in [11] makes clever use of Shearer’s lemma (Proposition 1).

**Theorem** **1** (Kahn 2001, [11]). *If G is a bipartite d-regular graph with n vertices, then*

(22) $$\begin{array}{c} \left|L(G)\right|\leq {\left(2^{d+1}-1\right)}^{\frac{n}{2d}}. \end{array}$$

*Furthermore, if n is an even multiple of d, then the upper bound in the right side of* (22) *is tight, and it is obtained by a disjoint union of $\frac{n}{2d}$ complete d-regular bipartite graphs $\left(K_{d,d}\right)$.*

Kahn’s result was later extended by Zhao [23] for a general *d*-regular graph via a brilliant combinatorial reduction to the setting of *d*-regular bipartite graphs, which proved Conjecture 4.1 in [11].

**Theorem** **2** (Zhao 2010, [23]). *The upper bound on the number of independent sets in* (22) *continues to hold for all d-regular graphs with n vertices.*

Recently, Sah et al. [25] proved Kahn’s conjecture (Conjecture 4.2 in [11], which was made eighteen years earlier) for an upper bound on the number of independent sets in a general undirected graph with no isolated vertices. The proof in [25] is combinatorial, and it extends the result in Theorem 2 as follows.

**Theorem** **3** (Sah et al. 2019, [25]). *Let G be an undirected graph without isolated vertices or multiple edges connecting any pair of vertices. Let $d_{v}$ denote the degree of a vertex v∈V(G). Then,*

(23) $$\begin{array}{c} \left|L(G)\right|\leq \prod_{\left(u,v)\in E(G\right)}{\left(2^{d_{u}}+2^{d_{v}}-1\right)}^{\frac{1}{d_{u}d_{v}}} \end{array}$$

*with an equality if G is a disjoint union of complete bipartite graphs.*

Let $K_{d_{u},d_{v}}$ be a complete bipartite graph where the degrees of its left and right vertices are equal to $d_{u}$ and $d_{v}$, respectively. Then, the number of independent sets in such a complete bipartite graph is equal to

(24) $$\begin{array}{c} \left|L(K_{d_{u},d_{v}})\right|=2^{d_{u}}+2^{d_{v}}-1 \end{array}$$

since every subset of the left vertices, as well as every subset of the right vertices, forms an independent set of $K_{d_{u},d_{v}}$; on the other hand, any subset which contains both left and right vertices is not an independent set (since the bipartite graph $K_{d_{u},d_{v}}$ is complete). Note that the substraction by 1 in the right side of (24) is because, in the counting of the number of subsets of left vertices ($2^{d_{v}}$) or right vertices ($2^{d_{u}}$), the empty set is counted twice. Hence, (23) can be rewritten in an equivalent form as

(25) $$\begin{array}{c} \left|L(G)\right|\leq \prod_{(u,v)\in EG}{\left|L(K_{d_{u},d_{v}})\right|}^{\frac{1}{d_{u}d_{v}}}. \end{array}$$

Since $E\left(K_{d_{u},d_{v}}\right)=d_{u}d_{v}$, it follows that the bound in (23) (or (25)) is achieved by the complete bipartite graph $K_{d_{u},d_{v}}$. More generally, the bound is achieved by a disjoint finite union of such complete bipartite graphs, since the number of independent sets in a disjoint union of graphs is equal to the product of the number of independent sets in each of these component graphs.

For the extension of the validity of Theorem 1 to Theorem 2, obtained by relaxing the requirement that the graph is bipartite, the following inequality was introduced by Zhao for every finite graph *G* (Lemma 2.1 in [23]):

(26) $$\begin{array}{c} {\left|L(G)\right|}^{2}\leq \left|L(G\times K_{2})\right|, \end{array}$$

which relates the number of independent sets in a graph to the number of independent sets in the bipartite double cover of this graph.

The transition from Theorem 1 to Theorem 2, as introduced in [23], is a one-line proof. Let *G* be a *d*-regular graph with *n* vertices, then $G\times K_{2}$ is *d*-regular bipartite graph with 2n vertices. Hence, (22) and (26) give

L(G)2≤L(G×K2)(27)≤(2d+1−1)2n2d,

and taking the square root of the leftmost and rightmost sides of (27) implies that (22) continues to hold even when the regular graph *G* is not necessarily bipartite.

## 3. Scientific Merit and Contributions of the Present Work

After introducing Shearer’s lemma (Proposition 1) and Theorems 1–3, we address the scientific merit and contributions of the present work in more detail (in comparison to the introduction in Section 1).

Theorem 3 was recently proved in [25] (see also [26]) for general graphs, without relying on information theory. The motivation of our work is rooted in the following sentences from p. 174 in [25]:

*Kahn’s proof [11] of the bipartite case of Theorem 1 made clever use of Shearer’s entropy inequality [2]. It remains unclear how to apply Shearer’s inequality in a lossless way in the irregular case, despite previous attempts to do so, e.g., Section 3 in [13] and Section 5.C in [14]*.

The present paper gives an information-theoretic proof of Theorem 3 in a setting where the bipartite graph is regular on one side (i.e., the vertices on the other side of the bipartite graph can be irregular, and have arbitrary degrees). Its contributions are as follows:

- Section 4 provides a (non-trivial) extension of the proof of (22), from regular bipartite graphs [11] to general bipartite graphs. This leads to an upper bound on the number of independent sets, which is in general looser than the bound in (23) (or its equivalent form in (25)). However, for the family of bipartite graphs that are regular on one side of the graph, our bound in (72) coincides with the bound in (23).
  The main deviation from Kahn’s information-theoretic proof in [11] is that here we allow the bipartite graph to be irregular. This generalization is not trivial in the sense that it requires a more careful analysis and a slightly more complicated version of Shearer’s Lemma (see Remark 1). It should be noted, however, that the suggested proof follows the same recipe of Kahn’s proof in [11], with some further complications that arise from the non-regularity of the bipartite graphs;
- A variant of the proof of Zhao’s inequality (26) (see Section 2 in [23]) is provided in Section 5.

It is interesting to note that the observation that (23) can be extended from (undirected) bipartite graphs to general graphs, by utilizing (26), was made in Lemma 3 in [24]. However, a computer-assisted proof of (23) was restricted there to graphs whose maximal degrees are at most 5 (see Theorem 2 in [24]).

## 4. An Information-Theoretic Proof of Theorem 3 for a Family of Bipartite Graphs

The core of the proof of Theorem 3 is proving (23) for an undirected bipartite graph. We provide an extension of the entropy-based proof by Kahn [11] from bipartite *d*-regular graphs to general bipartite graphs, and then we prove (23) for the family of bipartite graphs that are regular on one side. As is explained in Section 3, the proof in the present section follows the same recipe of Kahn’s proof in [11], with some complications that arise from the non-regularity of the bipartite graphs. The following proof deviates from the proof in [11] at its starting point, by a proper adaptation of the proof technique to the general setting of irregular bipartite graphs, followed by a slightly more complicated usage of Shearer’s lemma and a more involved analysis.

First consider a general bipartite graph *G* with a number of vertices |V(G)|=n, where none of its vertices is isolated. Label them by the elements of [1:n]. Let L and R be the vertices of the two types in V(G) (called, respectively, the left and right vertices in *G*), so V(G)=L∪R is a disjoint union. Let $D_{L}$ and $D_{R}$ be, respectively, the sets of all possible degrees of vertices in L and R. For all $d\in D_{L}$, let $L_{d}$ be the set of vertices in L with degree *d*, and let $R_{d}$ be the set of vertices in R that are adjacent to vertices in $L_{d}$ (note that the vertices in $R_{d}$ are not necessarily those vertices in R with degree *d*, so the definitions of $L_{d}$ and $R_{d}$ differ, i.e., they are not similar up to the replacement of left vertices of degree *d* with right vertices of the same degree). Then,

(28) $$\begin{array}{c} L=\underset{d\in D_{L}}{⋃}\;L_{d},\;R=\underset{d\in D_{L}}{⋃}\;R_{d}, \end{array}$$

where the first equality in (28) is (by definition) a union of pairwise disjoint sets.

Let S∈L(G) be an independent set in *G*, which is selected uniformly at random from L(G), and let $X^{n}≜\left(X_{1},\ldots ,X_{n}\right)$ be given by

(29) $$\begin{array}{c} X_{i}≜𝟙\left\{i\in S\right\},\;i\in \left[1:n\right], \end{array}$$

so the binary random vector $X^{n}$ indicates which of the *n* vertices in V(G) belongs to the randomly selected independent set S. Since S is equiprobable in L(G), we have

(30) $$\begin{array}{c} H\left(X^{n}\right)=\log\;\left|L(G)\right|. \end{array}$$

Let $X_{L}={\left(X_{i}\right)}_{i\in L}$ and $X_{R}={\left(X_{i}\right)}_{i\in R}$ be used as a shorthand. Then,

(31)H(Xn)=H(XL,XR)(32)=H(XL)+H(XR|XL)(33)≤∑d∈DLH(XLd)+H(XR|XL)(34)≤∑d∈DLH(XLd)+∑d∈DLH(XRd|XL)(35)=∑d∈DLH(XLd)+H(XRd|XL),

where inequalities (33) and (34) hold by the subadditivity of the entropy, and due to (28). It should be noted that although the first summand in the right side of (35) is an entropy of $X_{L_{d}}$, the conditioning on $X_{L}$ (rather than just on $X_{L_{d}}$) in the second term leads to a stronger upper bound on $H(X^{n})$ (since $L_{d}\subseteq L$, and conditioning reduces the entropy). This is essential for the continuation of the proof (see (37)).

We next upper bound the two summands in the right side of (35), starting with the conditional entropy. By invoking the subadditivity property of the entropy, for every $d\in D_{L}$,

(36) $$\begin{array}{c} H\left(X_{R_{d}}|\;X_{L}\right)\leq \sum_{r\in R_{d}}H\left(X_{r}|\;X_{L}\right). \end{array}$$

For every $r\in R_{d}$, let N(r) be the set of all the vertices that are adjacent to the vertex *r*. Since the graph *G* is bipartite, we have N(r)⊆L (but, in general, $N(r)⊈L_{d}$), and consequently

(37) $$\begin{array}{c} H\left(X_{r}|\;X_{L}\right)\leq H\left(X_{r}|\;X_{N(r)}\right). \end{array}$$

Combining (36) and (37) gives

(38) $$\begin{array}{c} H\left(X_{R_{d}}|\;X_{L}\right)\leq \sum_{r\in R_{d}}H\left(X_{r}|\;X_{N(r)}\right). \end{array}$$

For $r\in R_{d}$, let

(39) $$\begin{array}{c} Q_{r}≜𝟙\left\{S\cap N(r)=\emptyset \right\} \end{array}$$

be the indicator function of the event where none of the vertices that are adjacent (in *G*) to the vertex *r* are included in the (randomly selected) independent set S. Then,

(40) $$\begin{array}{c} H\left(X_{r}|\;X_{N(r)}\right)\leq H\left(X_{r}|\;Q_{r}\right), \end{array}$$

since the random vector $X_{N(r)}$ indicates which of the indices i∈N(r) are included in S, whereas the binary random variable $Q_{r}$ only indicates if there is such an index. Consequently, (38) and (40) imply that

(41) $$\begin{array}{c} H\left(X_{R_{d}}|\;X_{L}\right)\leq \sum_{r\in R_{d}}H\left(X_{r}|\;Q_{r}\right). \end{array}$$

For the binary random variable $Q_{r}$, let

(42) $$\begin{array}{c} q_{r}≜P\left[Q_{r}=1\right]. \end{array}$$

By (39), $Q_{r}=0$ if and only if S∩N(r)≠∅, which implies that r∉S, since there is a vertex in N(r) that belongs to the independent set S. Therefore, if $Q_{r}=0$, then $X_{r}=0$ (see (29)), so

(43) $$\begin{array}{c} H(X_{r}|\;Q_{r}=0)=0. \end{array}$$

If $Q_{r}=1$, then $X_{r}\in \left\{0,1\right\}$ and it is also equiprobable (the latter holds since given $Q_{r}=1$, the independent set S is uniformly distributed over all the independent sets in L(G) that do not include any neighbor of the vertex *r*, so the vertex *r* can be either removed from or added to such an independent set, while still giving an independent set that does not include any neighbor of *r*). Hence,

(44) $$\begin{array}{c} H(X_{r}|\;Q_{r}=1)=1. \end{array}$$

Hence, from (42) to (44),

H(Xr|Qr)=qrH(Xr|Qr=1)+(1−qr)H(Xr|Qr=0)(45)=qr,

and the combination of (41) and (45) yields

(46) $$\begin{array}{c} H\left(X_{R_{d}}|\;X_{L}\right)\leq \sum_{r\in R_{d}}q_{r}. \end{array}$$

We next upper bound $H(X_{L_{d}})$, which is the first summand in the right side of (35), and here Shearer’s lemma (see Proposition 1) comes into the picture. Since, by definition, $R_{d}$ is the set of the vertices that are connected to the subset $L_{d}$ of the degree-*d* vertices in L, and N(r) is the set of vertices in L that are connected to a vertex $r\in R_{d}$ in the bipartite graph *G*, then it follows that every vertex in $L_{d}$ belongs to at least *d* of the subsets ${\left\{N(r)\right\}}_{r\in R_{d}}$. Hence, by Shearer’s lemma (in light of Remark 1, this also holds regardless of the fact that, for $r\in R_{d}$, the set N(r) is not necessarily a subset of $L_{d}$),

(47) $$\begin{array}{c} H\left(X_{L_{d}}\right)\leq \frac{1}{d}\sum_{r\in R_{d}}H\left(X_{N(r)}\right). \end{array}$$

The binary random variable $Q_{r}$ is a deterministic function of the random vector $X_{N(r)}$ since, from (29) and (39), $Q_{r}=1$ if and only if all the entries of $X_{N(r)}$ are equal to 0. Consequently, for all $r\in R_{d}$,

(48)H(XN(r))=H(XN(r),Qr)(49)=H(Qr)+H(XN(r)|Qr)(50)=Hb(qr)+H(XN(r)|Qr),

where the equality in (50) follows from (2) and (42). Next, from (42),

(51) $$\begin{array}{c} H\left(X_{N(r)}|\;Q_{r}\right)=q_{r}H\left(X_{N(r)}|\;Q_{r}=1\right)+\left(1-q_{r}\right)H\left(X_{N(r)}|\;Q_{r}=0\right). \end{array}$$

If $Q_{r}=1$, then $X_{N(r)}$ is a vector of zeros, so

(52) $$\begin{array}{c} H(X_{N(r)}|\;Q_{r}=1)=0. \end{array}$$

Otherwise, if $Q_{r}=0$, then $X_{i}=1$ for at least one element i∈N(r); since $|N(r)|=d_{r}$ is the degree of the vertex *r* (by assumption, there are no multiple edges connecting any pair of vertices), it follows that the vector $X_{N(r)}\in {\left\{0,1\right\}}^{d_{r}}$ cannot be the zero vector, so

(53) $$\begin{array}{c} H\left(X_{N(r)}|\;Q_{r}=0\right)\leq \log\left(2^{d_{r}}-1\right). \end{array}$$

Combining (48)–(53) gives

(54) $$\begin{array}{c} H\left(X_{N(r)}\right)\leq H_{b}\left(q_{r}\right)+\left(1-q_{r}\right)\log\left(2^{d_{r}}-1\right), \end{array}$$

and, from (47) and (54), the following upper bound on the first summand in the right side of (35) holds:

(55) $$\begin{array}{c} H\left(X_{L_{d}}\right)\leq \frac{1}{d}\sum_{r\in R_{d}}\left\{H_{b}\left(q_{r}\right)+\left(1-q_{r}\right)\log\left(2^{d_{r}}-1\right)\right\}. \end{array}$$

Consequently, combining (31)–(35), (46) and (55) implies that

(56)H(Xn)≤∑d∈DLH(XLd)+H(XRd|XL)(57)≤∑d∈DL∑r∈Rdqr+1d∑r∈RdHb(qr)+(1−qr)log(2dr−1)(58)=∑d∈DL1d∑r∈RdHb(qr)+(1−qr)log(2dr−1)+qrlog(2d)(59)=∑d∈DL1d∑r∈RdHb(qr)+qrlog2d2dr−1+log(2dr−1).

Since $q_{r}\in \left[0,1\right]$ for $r\in R_{d}$, we next maximize an auxiliary function $f_{r}:\left[0,1\right]\to R$, defined as

(60) $$\begin{array}{c} f_{r}\left(x\right)≜H_{b}\left(x\right)+x\log\left(\frac{2^{d}}{2^{d_{r}}-1}\right),\;x\in \left[0,1\right], \end{array}$$

in order to obtain an upper bound on the right side of (59) which is independent of $\left\{q_{r}\right\}$. By (2), setting the first derivative of the concave function $f_{r}\left(\cdot \right)$ to zero gives the equation

(61) $$\begin{array}{c} \log\left(\frac{1-x}{x}\right)+\log\left(\frac{2^{d}}{2^{d_{r}}-1}\right)=0, \end{array}$$

whose solution is given by

(62) $$\begin{array}{c} x=\frac{2^{d}}{2^{d}+2^{d_{r}}-1}. \end{array}$$

Consequently, it follows from (56)–(60) and (62) that

(63)H(Xn)≤∑d∈DL1d∑r∈Rdfr(qr)+log(2dr−1)(64)≤∑d∈DL1d∑r∈Rdfr2d2d+2dr−1+log(2dr−1)

and the calculation of the term in the inner sum in the right side of (64) gives

fr2d2d+2dr−1+log(2dr−1)(65)=Hb2d2d+2dr−1+2d2d+2dr−1log2d2dr−1+log(2dr−1)=−2d2d+2dr−1log2d2d+2dr−1−2dr−12d+2dr−1log2dr−12d+2dr−1(66)+2d2d+2dr−1log2d2dr−1+log(2dr−1)=−2d2d+2dr−1log2d2d+2dr−1−log2d2dr−1(67)−2dr−12d+2dr−1log2dr−12d+2dr−1+log(2dr−1)=−2d2d+2dr−1log2dr−12d+2dr−1−2dr−12d+2dr−1log2dr−12d+2dr−1(68)+log(2dr−1)(69)=−log2dr−12d+2dr−1+log(2dr−1)(70)=log2d+2dr−1,

where (65) and (66) hold, respectively, by (60) and (2). Substituting the equality in (70) into the upper bound on the entropy in the right side of (64), together with (30), gives

(71) $$\begin{array}{cc} \log\;\left|L(G)\right| & \leq \sum_{d\in D_{L}}\left\{\frac{1}{d}\sum_{r\in R_{d}}\log\left(2^{d}+2^{d_{r}}-1\right)\right\}, \end{array}$$

which, by exponentiation of both sides of (71), gives

(72) $$\begin{array}{c} \left|L(G)\right|\leq \prod_{d\in D_{L}}\prod_{r\in R_{d}}{\left(2^{d}+2^{d_{r}}-1\right)}^{\frac{1}{d}}. \end{array}$$

The upper bound in the right-hand side of (72) is, in general, looser than the bound in Theorem 3. Indeed, to clarify this point, let $\Gamma_{d,d^{\prime }}$ denote the fraction of vertices in $R_{d}$ with a degree $d^{\prime }\in D_{R}$. Then,

(73)∏d∈DL∏r∈Rd(2d+2dr−1)1d=∏d∈DL∏d′∈DR(2d+2d′−1)|Rd|Γd,d′d(74)=∏d∈DL∏d′∈DR(2d+2d′−1)1dd′d′|Rd|Γd,d′(75)≥∏(u,v)∈E(G)2du+2dv−11dudv,

where (73) holds since, for all $d\in D_{L}$, the number of vertices in $R_{d}$ with degree $d^{\prime }\in D_{R}$ is equal to $|R_{d}|\;\Gamma_{d,d^{\prime }}$; finally, (75) holds since the number of edges e=(u,v)∈E(G) that connect the left vertices of degree *d* and right vertices of degree $d^{\prime }$ is less than or equal to $d^{\prime }\;\left|R_{d}\right|\;\Gamma_{d,d^{\prime }}$ (since $d^{\prime }$ edges emanate from each such right vertex, but these edges are not necessarily connected to left vertices of degree *d*). In light of this explanation, there is however an interesting case where the upper bound in the right side of (72) and the bound in Theorem 3 coincide.

Let *G* be a bipartite graph that is *d*-regular on one side (i.e, one type of its vertices have a fixed degree *d*, and the other type of vertices may be irregular with arbitrary degrees). Without any loss of generality, one can assume that the left vertices are *d*-regular (as, otherwise, the graph can be flipped without affecting its independent sets, and also the bound in Theorem 3 is symmetric in the degrees $d_{u}$ and $d_{v}$). In this setting, $L_{d}=L$ and $R_{d}=R$ (recall that, by assumption, there are no isolated vertices). Consequently, the right side of (72) is specialized to

(76) $$\begin{array}{c} \left|L(G)\right|\leq \prod_{r\in R}{\left(2^{d}+2^{d_{r}}-1\right)}^{\frac{1}{d}}. \end{array}$$

Since there are exactly $d_{r}$ edges connecting each vertex r∈R with vertices in L, and (by the latter assumption) all of the left vertices in L are of a *fixed degree* *d*, it follows that in this setting, the right side of (76) can be rewritten in the form

(77)∏r∈R2d+2dr−11d=∏r∈R2d+2dr−11ddrdr(78)=∏(u,v)∈E(G)2du+2dv−11dudv,

which, indeed, shows that the right side of (72) and the bound in Theorem 3 coincide for bipartite graphs that are regular on one side of the graph (without restricting the other side).

## 5. A Variant of the Proof of Zhao’s Inequality

This section suggests a variant of the proof of Zhao’s Inequality in (26) (see Lemma 2.1 in [23]). Although it is somewhat different from the one in [23], this forms in essence a reformulation of Zhao’s proof, which is provided here for completeness.

Let *G* be a finite graph, and let $\left|V(G)\right|=n$. Label the vertices in the left and right sides of the bipartite graph $G\times K_{2}$ (i.e., the bipartite double cover of *G*) by ${\left\{\left(i,0\right)\right\}}_{i=1}^{n}$ and ${\left\{\left(i,1\right)\right\}}_{i=1}^{n}$, respectively.

Choose, independently and uniformly at random, two independent sets $S_{0},S_{1}\in L(G)$. For i∈[1:n], let $X_{i},Y_{i}\in \left\{0,1\right\}$ be random variables defined as $X_{i}=1$ if and only if $i\in S_{0}$, and $Y_{i}=1$ if and only if $i\in S_{1}$. Then, by the statistical independence and equiprobable selection of the two independent sets from L(G), we have

(79)H(Xn,Yn)=H(Xn)+H(Yn)(80)=2logL(G),

where (79) holds since the random vectors $X^{n}$ and $Y^{n}$ are statistically independent (by construction), and (80) holds since both $X^{n}$ and $Y^{n}$ have an equiprobable distribution over a set whose cardinality is $\left|L(G)\right|$.

Consider the following set of vertices in $G\times K_{2}$:

(81)S≜S0×{0}⋃S1×{1}(82)=⋃i∈S0,j∈S1(i,0),(j,1).

The set S is not necessarily an independent set in $G\times K_{2}$; indeed, $\left((i,0),(j,1)\right)\in E(G\times K_{2})$ for all $i\in S_{0}$ and $j\in S_{1}$ for which (i,j)∈E(G) (see (21)). We next consider all (i,j)∈E(G), such that $X_{i}=Y_{j}=1$. To that end, fix an ordering of all the $2^{n}$ subsets of V(G), and let T∈V(G) be the first subset in this particular ordering which includes exactly one endpoint of each edge (i,j)∈E(G) for which $X_{i}=Y_{j}=1$. Consider the following replacements:

- If (i,0)∈S and i∈T, then (i,0) is replaced by (i,1);
- Likewise, if (j,1)∈S and j∈T, then (j,1) is replaced by (j,0).

Let $\tilde{S}$ be the set of new vertices after these possible replacements. Then, $\tilde{S}\in L(G\times K_{2})$, since all adjacent vertices in S are no longer connected in $\tilde{S}$. Indeed, there is no way that after (say) a vertex (i,0) is replaced by (i,1), there is another replacement of a vertex (j,1) by (j,0), for some *j* such that (i,j)∈E(G); otherwise, that would mean that T contains both *i* and *j*, which is impossible by construction.

Similarly to the way in which $X^{n},Y^{n}\in {\left\{0,1\right\}}^{n}$ were defined, let ${\tilde{X}}^{n},{\tilde{Y}}^{n}\in {\left\{0,1\right\}}^{n}$ be defined such that, for all i∈[1:n], ${\tilde{X}}_{i}=1$ if and only if $\left(i,0\right)\in \tilde{S}$, and ${\tilde{Y}}_{i}=1$ if and only if $\left(i,1\right)\in \tilde{S}$. The mapping from $\left(X^{n},Y^{n}\right)$ to $\left({\tilde{X}}^{n},{\tilde{Y}}^{n}\right)$ is injective. Indeed, it is shown to be injective by finding all indices (i,j)∈E(G) such that ${\tilde{X}}_{i}={\tilde{X}}_{j}=1$ or ${\tilde{Y}}_{i}={\tilde{Y}}_{j}=1$, finding the first subset T∈V(G) according to our previous fixed ordering of the $2^{n}$ subsets of V(G) that includes exactly one endpoint of each such edge (i,j)∈E(G), and performing the reverse operation to return to $X^{n}$ and $Y^{n}$ (e.g., if (i,j)∈E(G), ${\tilde{X}}_{i}={\tilde{X}}_{j}=1$ and i∈T while j∉T, then ${\tilde{X}}_{i}=1$ is transformed back to $Y_{i}=1$, and ${\tilde{X}}_{j}=1$ is transformed back to $X_{j}=1$). Consequently, we get

(83)H(Xn,Yn)=H(X˜n,Y˜n)(84)≤logL(G×K2),

where (83) holds by the injectivity of the mapping from $\left(X^{n},Y^{n}\right)$ to $\left({\tilde{X}}^{n},{\tilde{Y}}^{n}\right)$, and (84) holds since $\tilde{S}$ is an independent set in $G\times K_{2}$, which implies that $\left({\tilde{X}}^{n},{\tilde{Y}}^{n}\right)$ can get (at most) $\left|L(G\times K_{2})\right|$ possible values (by definition, there is a one-to-one correspondence between $\tilde{S}$ and $\left({\tilde{X}}^{n},{\tilde{Y}}^{n}\right)$). Combining (79), (80), (83) and (84) gives

(85) $$\begin{array}{c} 2\log\;\left|L(G)\right|\leq \log\;\left|L\left(G\times K_{2}\right)\right|, \end{array}$$

which gives (26) by exponentiation of both sides of (85).

## Data Availability

Data sharing not applicable.
